# Role of Estrogens in the Size of Neuronal Somata of Paravaginal Ganglia in Ovariectomized Rabbits

**DOI:** 10.1155/2017/2089645

**Published:** 2017-02-21

**Authors:** Laura G. Hernández-Aragón, Verónica García-Villamar, María de los Ángeles Carrasco-Ruiz, Leticia Nicolás-Toledo, Arturo Ortega, Estela Cuevas-Romero, Margarita Martínez-Gómez, Francisco Castelán

**Affiliations:** ^1^Centro Tlaxcala de Biología de la Conducta, Universidad Autónoma de Tlaxcala (UATx), Tlaxcala, TLAX, Mexico; ^2^Departamento de Toxicología, Centro de Investigación y de Estudios Avanzados del Instituto Politécnico Nacional, Ciudad de México, CDMX, Mexico; ^3^Departamento de Biología Celular y Fisiología, Instituto de Investigaciones Biomédicas, Universidad Nacional Autónoma de México (UNAM), Unidad Foránea Tlaxcala, Tlaxcala, TLAX, Mexico

## Abstract

We aimed to determine the role of estrogens in modulating the size of neuronal somata of paravaginal ganglia. Rabbits were allocated into control (C), ovariectomized (OVX), and OVX treated with estradiol benzoate (OVX + EB) groups to evaluate the neuronal soma area; total serum estradiol (E2) and testosterone (T) levels; the percentage of immunoreactive (ir) neurons anti-aromatase, anti-estrogen receptor (ER*α*, ER*β*) and anti-androgen receptor (AR); the intensity of the immunostaining anti-glial cell line-derived neurotrophic factor (GDNF) and the GDNF family receptor alpha type 1 (GFR*α*1); and the number of satellite glial cells (SGCs) per neuron. There was a decrease in the neuronal soma size for the OVX group, which was associated with low T, high percentages of aromatase-ir and neuritic AR-ir neurons, and a strong immunostaining anti-GDNF and anti-GFR*α*1. The decrease in the neuronal soma size was prevented by the EB treatment that increased the E2 without affecting the T levels. Moreover, there was a high percentage of neuritic AR-ir neurons, a strong GDNF immunostaining in the SGC, and an increase in the SGCs per neuron. Present findings show that estrogens modulate the soma size of neurons of the paravaginal ganglia, likely involving the participation of the SGC.

## 1. Introduction

The pelvic plexus supplies most of the autonomic innervation that receives the lower urogenital tract in vertebrates [1]. Childbirth has been postulated as a source of variability of the pelvic plexus that may lead to some urological and gynecological symptoms [2–4]. The impact of changes in steroid hormone levels during pregnancy and postpartum on the lower urogenital tract (LUT) has been underestimated despite the widespread expression of their receptors in pelvic neurons [5]. Estrogen actions are particularly interesting because they support therapies used to alleviate some urological symptoms in postmenopausal women [6].

The estrogenic status influences the size of neuronal somata of pelvic ganglia in female rats and rabbits [7, 8]. In this regard, it has been hypothesized that the increase in the serum estradiol (E2) levels at term of pregnancy is involved in the recovery of the soma size of paravaginal neurons during the postpartum period in rabbits [7, 9]. Under physiological conditions, however, such a peak of serum E2 occurs concomitantly to an increase in serum testosterone (T) levels [10]. Androgens modulate the morphology of pelvic neurons as showed for male rats, which could require their conversion into estrogens by the cytochrome P450 aromatase (hereafter aromatase) [5, 11, 12]. Estrogen and androgen actions involved in the morphological plasticity of pelvic neurons have been linked to the signaling of neurotrophic factors including ligands from the glial cell line-derived neurotrophic factor (GDNF) family (GFLs) [5, 11–17]. The GFLs differentially interact with members of the GDNF family receptors (GFR*α*1–4), having the GFR*α*1 the greatest affinity for the GDNF [18].

The female rabbit is a reflex ovulator that exhibits constant serum E2 levels unless mating occurs. In accordance with some reports, ovariectomy has little or no effect on E2 levels in this species [19–21]. This may be related to an increase in the extragonadal aromatization that could impair concurrent actions mediated by circulating androgens [20, 22]. Taking into account that pelvic floor tissues of some female mammals express aromatase [20, 23], it is reasonable to expect that the locally synthesized estrogens and androgens are also relevant for the morphology of pelvic neurons.

On this framework, the imbalance between serum E2 and T levels because of ovariectomy could affect the morphology of paravaginal neurons in rabbits. Since E2 is able to restore the size of pelvic neurons in female rats [8], the extent in which estrogens could revert the effect of ovariectomy was approached herein.

The present study aimed therefore to determine the role of estrogens in modulating the size of neuronal somata of paravaginal ganglia in control (C), ovariectomized (OVX), and OVX treated with estradiol benzoate (OVX + EB) rabbits. Furthermore, we evaluated the total serum E2 levels and the expression of estrogen receptors (ER*α*, ER*β*) in paravaginal neurons. Moreover, total serum T levels and androgen receptor (AR) and aromatase expression in paravaginal neurons were also estimated. Because estradiol modulates the expression of the GDNF and GFR*α*1 [24–26], a plausible relationship between estrogens and the expression of GDNF and GFR*α*1 was also evaluated.

## 2. Material and Methods

Unless otherwise is stated, chemicals were purchased from Sigma-Aldrich, México.

### 2.1. Animals

Twenty-one six-month-old Chinchilla-breed female rabbits* (Oryctolagus cuniculus)* were housed in individual stainless-steel cages and kept at 20 ± 2°C under artificial lighting conditions (L : D 16 : 8, lights on at 0600 h) in which it is considered that rabbits are at an early proestrous phase [27]. They were daily provided with pellet food (Conejina, Purina) and had continuous access to water. The Ethics Committee from the Centro Tlaxcala de Biología de la Conducta, Universidad Autónoma de Tlaxcala, approved all of the following experimental procedures that were in agreement with the National Guide for the Production, Care and Use of Laboratory Animals (Norma Oficial Mexicana NOM-062-200-1999, Mexico).

Rabbits were allocated randomly in three groups, C (*n* = 6), OVX (*n* = 6), and OVX treated with EB (OVX + EB, *n* = 6). Bilateral ovariectomy was done using xylazine (20 mg/Kg, i.p., Pisa) and ketamine (20 mg/Kg, i.p., Pisa) as anesthetics. Under same dosage of these anesthetics and after 3.5 months, OVX rabbits were implanted in the base of the dorsal neck (intrascapular area) with empty Silastic capsules (20 mm long, 3.18 mm O.D., 1.98 mm I.D.; Dow Corning Corporation) sealed with wooden plugs (group OVX), or Silastic capsules containing ~70 mg of EB (17*β*-estradiol-3-benzoate; OVX + EB group). After 1.5 months, OVX and OVX + EB rabbits were euthanized with an overdose of sodium pentobarbital (60 mg/kg, i.p., Pisa). Virgin rabbits of the C group were not manipulated and killed when they reached the same age of OVX and OVX + EB rabbits (~11 months old).

### 2.2. Histology

At the end of the experimental period, the pelvic vagina was excised, washed in saline, immersed in Bouin-Duboscq fixative, and embedded in paraplast X-tra. Tissues were cut on a microtome (Leica) to obtain 7 *μ*m transverse sections. Slides were separated in four series. One of them was stained with Masson's trichrome, covered with mounting medium (Cytoseal 60, Richard-Allan Scientific) and a coverslip, and observed under light microscopy by using an Axio Imager A1 microscope (Carl Zeiss). Images were acquired with a digital camera (ProgRes® CT5, Jenoptik) with a resolution of 5.1 megapixels.

### 2.3. Morphometry

Paravaginal ganglia and neuron profiles were analyzed in a ~1.4 mm length segment of the pelvic vagina (about 20% of its whole length) as described elsewhere [7]. The urethral opening was set as reference point to sample ten cranial sections and other ten caudal sections, all of them chosen one every 10th 7 *μ*m section evaluating the first section of each series. Therefore, twenty sections per rabbit (120 sections per group) were examined. Images were analyzed using the program AxioVision Rel 4.6 (Carl Zeiss) to measure the total area covered by ganglia per field (ganglionic area), the number of ganglionic neurons, and the neuronal soma area. Neuronal profiles whose nuclei were clearly visible were counted and their area was measured. To obtain the number of neurons along the pelvic vagina segment, each raw neuron counted was multiplied by 10 to correct for uncounted sections and the Abercrombie method was used for correcting split nuclei [7, 28]. The number of satellite glial cells (SGCs) per neuron was estimated in two Masson-stained sections per rabbit (one cranial and another caudal to the urethral opening having a separation distance ~168 *μ*m) by counting the nuclei of the SGC associated as reported by other authors [29]. Only neuronal somata with visible nucleus were included for this analysis.

### 2.4. Serum E2 and T Levels

Total serum E2 levels were measured by a commercial laboratory (Carpermor S.A. de C.V.) using a chemiluminescent microparticle immunoassay (Architect Estradiol, Abbot); the origin of samples was blinded to the personnel. Serum T was measured by using commercially available EIA kits (Cayman Chemical Company) as described elsewhere [27]. Moreover, the log E2/T ratio was calculated to estimate the extent in which extragonadal aromatization could explain the recovery of serum E2 levels [20, 30].

### 2.5. Aromatase Expression

The left ovary and the pelvic vagina were excised from 3 rabbits of the C group (distinct of those in which the vaginal tissue was driven to the histological analysis). They were immediately frozen and stored at −80°C until analyzed. Approximately 150 mg of ovarian and vaginal tissues for each rabbit was disrupted using an electronic homogenizer (TissueTearor, BioSpec Products, Inc.) in lysis buffer (20 mM tris-HCl pH 7.4, 100 mM glycine, 100 mM NaCl, 0.1% triton X-100, 1 mM phenylmethylsulfonyl fluoride, and 1 mM DL-dithiothreitol) added with Protease Inhibitor Cocktail. These total protein extracts were assessed by Western blot using the experimental procedure described elsewhere [20]. Equal amounts of protein (ovary, 50 *µ*g; vagina, 50 *µ*g) were denatured in Laemmli's sample buffer, resolved through 10% SDS-polyacrylamide gels, and electrotransferred to nitrocellulose membranes (Bio-Rad Laboratories Headquarters). After finishing, nitrocellulose membranes were stained with 0.3% Ponceau's red (Amresco) dissolved in 1% acetic acid to assess that similar amounts of proteins were loaded in each lane. Membranes were soaked in phosphate buffered saline (PBS; 0.16 mM NaH_2_PO_4_, 0.34 mM Na_2_HPO_4_, and 154 mM NaCl) added with 0.2% tween-20 (PBST) and incubated in 5% dried skimmed milk diluted in PBS for 1 h to block nonspecific protein binding sites. Membranes were incubated overnight at 4°C with the primary antibody (see Table 1) diluted with milk 1% in PBS followed by secondary antibodies (goat anti-rabbit IgG-HRP, sc-2004, Santa Cruz Biotechnology Inc.) for 2 h. Immunoreactive polypeptides were detected using a chemiluminescence kit (West Pico Signal, Thermo Scientific) and exposed to a chemiluminescent-signal analyzer (MyECL, Thermo Scientific). The expression of aromatase in paravaginal neurons from rabbits of the C, OVX, and OVX + EB groups was indirectly evaluated by immunohistochemistry (IHC) as described below.

### 2.6. Immunohistochemistry

The expressions of aromatase, ER*α* and ER*β*, AR, GDNF, GFR*α*1, and glial fibrillary acidic protein (GFAP) were analyzed by IHC using the experimental procedure described elsewhere [7]. Slides containing vaginal sections were deparaffinized and microwaved in 10 mM sodium citrate pH 6 to retrieve antigens. Endogenous peroxidases were quenched with 0.3% hydrogen peroxide diluted in PBS at room temperature. Slides were rinsed twice with PBS, and endogenous binding sites for secondary antibodies were blocked with 5% NGS diluted in PBS with 0.3% triton X-100 (PBSTx). Slides were incubated with the primary antibody diluted in PBSTx in a humidified chamber for 72 h at 4°C. The corresponding primary antibody (see Table 1) was diluted in PBSTx and slides were incubated in a humidified chamber during 72 h at 4°C. Subsequently, slides were incubated with secondary antibodies (see Table 1), washed with PBS, and the immunostaining was developed with the Vectastain ABC kit (Vector Labs). Afterwards, sections were counterstained with Mayer's hematoxylin; slides were covered with the mounting medium and a coverslip and observed under light microscopy using a Ni-NU microscope (Nikon) coupled to a digital camera with a resolution of 16.25 megapixels (DS-Ri2, Nikon). No staining was seen in sections incubated with the secondary antibody alone (data not shown).

### 2.7. Immunostaining Analyses

Approximately 30 neurons per section per rabbit were sampled from immunostained sections (observed at a 400x magnification under the Nikon microscope). The ratio of (cytoplasmic) aromatase-ir neurons to the total number of neurons per section was calculated and expressed as percentage. The classification of ER*α*- or ER*β*-immunoreactive (ir) neurons was based on the nuclear location for each immunostaining. The ratio of ER*α*- or ER*β*-ir neurons to the number of total (labeled and unlabeled) neurons per section was therefore calculated per rabbit and expressed as percentage. A similar procedure was done to estimate the percentage of nuclear AR-ir neurons. Due to the fact that the anti-AR used for the present experiments is useful to detect the presence of AR in axon and dendrites [31], the percentage of neuritic AR-ir neurons was also measured. Homologous sections of the cranial portion of pelvic vagina for the C, OVX, and OVX + EB groups were simultaneously processed to estimate qualitatively the GDNF- and GFR*α*1-immunoreactivity based on the intensity of each marker in neurons and SGC. The cytoplasmic GFAP immunostaining was used to identify SGC [32]. The ratio of GFAP-ir ensheathed neurons to the total number of neurons per section was calculated and expressed as percentage. The number of SGCs per neuron was estimated indirectly counting the peripheral nuclei adjoined to neurons in Masson-stained sections.

### 2.8. Data Analysis

Data are means ± standard error (SEM). One-way ANOVA was used to analyze the statistical difference (*P* ≤ 0.05) between groups. To assess the statistical difference (*P* < 0.05) between pairs of groups, Newman–Keuls tests were used as post hoc tests. Statistical tests were done using the program Prism 5 for Mac (GraphPad Software).

## 3. Results

### 3.1. Soma Size of Paravaginal Neurons

**Figure 1:**
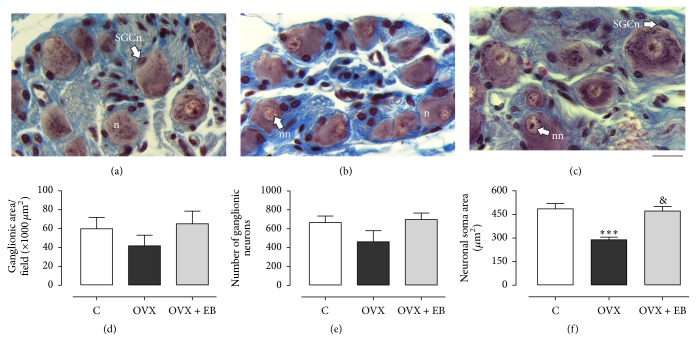
Neuronal soma size of paravaginal ganglia for the C (a), OVX (b), and OVX + EB (c) groups. (a–c) Representative photomicrographs of pelvic vaginal sections stained with Masson's trichrome. (d) Ganglionic area per field, (e) number of ganglionic neurons after Abercrombie's correction, and (f) neuronal soma area. Data are mean ± SEM (*n* = 6 per group). One-way ANOVA followed by Newman–Keuls tests were carried out to determine significant differences between groups. ^*∗∗∗*^*P* < 0.001 (compared to the C group); ^&^*P* < 0.001 (compared to the OVX group). n, neuron; nn, neuronal nucleus; SGCn, satellite glial cell nucleus. Bar, 20 *µ*m.

The soma size of neurons from the paravaginal ganglia was analyzed in Masson-stained sections (Figures 1(a)–1(c)). The ganglionic area (59820 ± 12100, 41810 ± 11210, 65180 ± 13350 *μ*m^2^;* F*_(2,15)_ = 0.9982, *P* = 0.3917; Figure 1(d)) and the number of ganglion neurons (664.2 ± 69.7, 461.6 ± 117.0, 696 ± 68;* F*_(2,15)_ = 2.083, *P* = 0.1591, Figure 1(e)) of the segment of pelvic vagina were similar between the C, OVX, and OVX + EB groups. In contrast, the soma area of paravaginal neurons was different between groups (C, 484.7 ± 33.5; OVX, 288.9 ± 17.1; OVX + EB, 471.1 ± 29.2 *µ*m^2^;* F*_(2,15)_ = 15.82, *P* = 0.0002). The post hoc tests showed that the neuronal soma size for the OVX group was smaller in comparison with the C and OVX + EB groups (Figure 1(f); *P* < 0.001). Those values for the C and OVX + EB groups were similar (*P* > 0.05).

### 3.2. Serum E2 and T Levels

**Figure 2:**
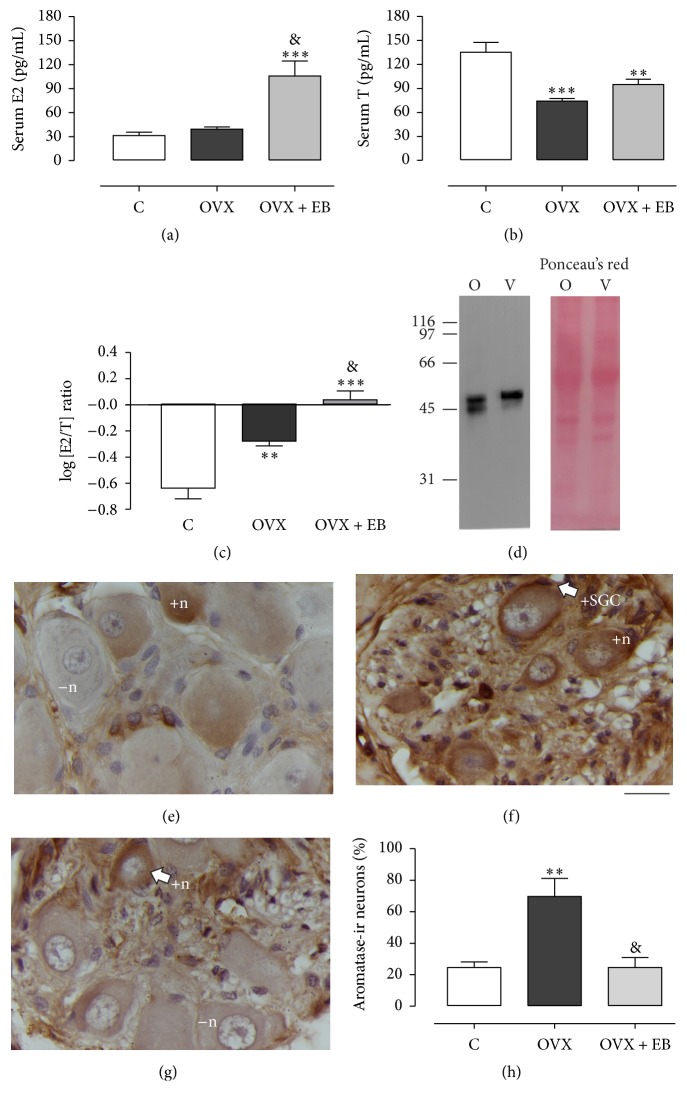
Serum concentrations of total estradiol (E2, (a)) and testosterone (T, (b)) vary between the C, OVX, and OVX + EB groups. (c) The ratio of E2 to T (as logarithm) was calculated to estimate the extent of extragonadal aromatization. Data are the mean ± SEM (*n* = 6 per group). (d) Aromatase expression in the ovary (O) and vagina (V) for control rabbits; Ponceau's Red staining was used to corroborate equal amounts of protein were loaded. Paravaginal neurons from the C (e), OVX (f), and OVX + EB (g) groups express aromatase as showed by immunohistochemistry. (h) Percentages of aromatase-ir neurons are means ± SEM (*n* = 6 per group). One-way ANOVA followed by Newman–Keuls post hoc tests were carried out to determine significant differences between groups. ^*∗∗*^*P* < 0.01 and ^*∗∗∗*^*P* < 0.001 (compared to the C group); ^&^*P* < 0.001 (compared to the OVX group). −n, negative neurons; +n, positive neurons; +SGC, positive satellite glial cell. Bar, 20 *µ*m.

Total serum E2 levels were different between the C, OVX, and OVX + EB groups (29.5 ± 2.7, 39.2 ± 2.8, 105.7 ± 18.7 pg/mL;* F*_(2,15)_ = 14.2, *P* = 0.003; Figure 2(a)). The results of post hoc tests indicated that the C and OVX groups had similar levels (*P* > 0.05). In contrast, the serum E2 levels for the OVX + EB group were higher than for those of the C and OVX groups (*P* < 0.001).

Total serum T levels were also different between the C, OVX, and OVX + EB groups (139.7 ± 13.9, 74.1 ± 3.2, 94.9 ± 6.5 pg/mL;* F*_(2,15)_ = 13.65, *P* = 0.0004; Figure 2(b)). The post hoc analysis revealed that total serum T levels for the OVX were lower in comparison with the C (*P* < 0.001) and OVX + EB (*P* < 0.01) groups. No differences between the T levels of the C and OVX + EB groups (*P* > 0.05) were observed.

The E2/T ratio (logarithm) changed between the C, OVX, and OVX + EB groups (−0.67 ± 0.07, −0.28 ± 0.04, 0.037 ± 0.07;* F*_(2,15)_ = 32.87, *P* > 0.0001; Figure 2(c)). The values for OVX and OVX + EB groups were higher (*P* < 0.001; *P* < 0.0001) than for that of the C group. The same was true for the comparison between the OVX and OVX + EB groups (*P* < 0.01).

### 3.3. Aromatase Expression

Western blot assays revealed the expression of aromatase in the vagina of control rabbits as supported by the presence of a major band around the expected molecular size of 55 kDa (Figure 2(d)). To examine the expression of aromatase in the paravaginal ganglia, a cytoplasmic immunostaining was observed in paravaginal neurons from the C, OVX, and OVX + EB groups (Figures 2(e)–2(g)). Moreover, an aromatase-ir sheath was observed in some SGC, particularly for the OVX group. The percentage of aromatase-ir neurons was different between the C, OVX, and OVX + EB groups (24.5 ± 3.4, 69.7 ± 11.5, 24.5 ± 6.2%;* F*_(2,15)_ = 11.04, *P* = 0.0011; Figure 2(h)). In contrast, the percentage of aromatase-ir neurons for the OVX group was higher than for the C (*P* < 0.01) and OVX + EB (*P* < 0.01) groups. No differences between the C and OVX + EB groups (*P* > 0.05) were observed.

### 3.4. ER Expression

**Figure 3:**
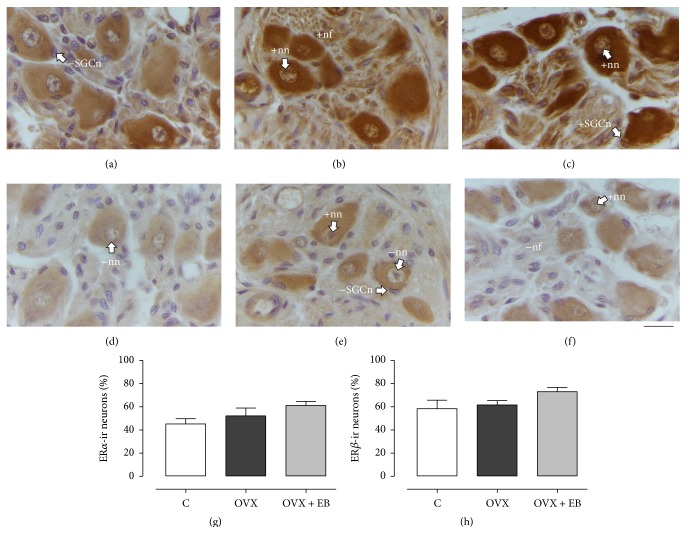
Expression of estrogen receptors (ER*α* and ER*β*) in paravaginal neurons of C (a, d), OVX (b, e), and OVX + EB (c, f) groups. Representative photomicrographs showing the nuclear ER*α*-ir (a–c) and ER*β*-ir neurons (d–f). The percentages of ER*α*-ir (g) and ER*β*-ir neurons (h) were similar between groups. Data are means ± SEM (*n* = 6 per group). One-way ANOVA followed by Newman–Keuls post hoc tests were carried out to determine significant differences between groups. +nf, positive neural fiber; +nn, positive neuronal nucleus; −nn, negative neuronal nucleus; +SGCn, positive satellite glial cell nucleus; −SGCn, negative satellite glial cell nucleus. Bar, 20 *µ*m.

The ER*α*- (Figures 3(a)–3(c)) and ER*β*-immunostaining (Figures 3(d)–3(f)) were observed at cytoplasm and nucleus of neurons of the paravaginal ganglia from the C, OVX, and OVX + EB groups. Sparse peripheral nuclei adjoined to paravaginal neurons were also ER*α*-ir. In comparison with the C group, a stronger ER*α* immunoreactivity *α* in the cytoplasm of neurons was observed for the OVX and OVX + EB groups. However, the percentage of ER*α*-ir neurons was similar between the three experimental groups (C, 45.2 ± 4.4; OVX, 52.1 ± 6.7; OVX + EB, 61.1 ± 3.5%;* F*_(2,15)_ = 2.421, *P* = 0.1227; Figure 3(g)). The same was true for the percentage of ER*β*-ir neurons (C, 58.4 ± 7.2; OVX, 61.6 ± 3.7; OVX + EB, 73 ± 3.8%;* F*_(2,15)_ = 2.177, *P* = 0.1428; Figure 3(h)).

### 3.5. AR Expression

**Figure 4:**
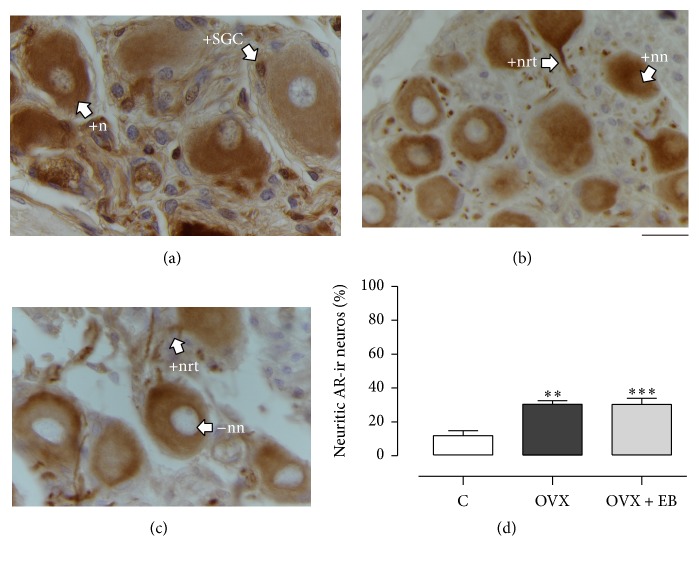
Expression of the androgen receptor (AR) in the paravaginal neurons of the C (a), OVX (b), and OVX + EB (c) groups. Representative photomicrographs showing the nuclear and neuritic AR immunoreactivity. (d) The percentage of neuritic AR-ir neurons is expressed as the mean ± SEM (*n* = 6 per group). One-way ANOVA followed by Newman–Keuls post hoc tests were carried out to determine significant differences between groups. ^*∗∗*^*P* < 0.01, ^*∗∗∗*^*P* < 0.001 (compared to the C group). +n, positive neuronal cytoplasm; +nn, positive neuronal nucleus; −nn, negative neuronal nucleus; +nrt, positive neurite; +SGC, positive satellite glial cell. Bar, 20 *µ*m.

The AR immunoreactivity was noticed at nucleus and cytoplasm of neurons from the paravaginal ganglia, including neurites, for the C, OVX, and OVX + EB groups (Figures 4(a)–4(c)). The AR-ir was also observed in other ganglionic components as putative SGC and nerve bundles. The percentage of nuclear AR-ir neurons was similar between the three groups (C, 49.6 ± 4; OVX, 44.9 ± 5.7; OVX + EB, 38.6 ± 7.9%;* F*_(2,15)_ = 0.8261, *P* = 0.4567). In contrast, the percentage of neuritic AR-ir neurons was different (C, 11.8 ± 3; OVX, 30.3 ± 2.2; OVX + EB, 30.2 ± 3.6%;* F*_(2,15)_ = 12.46, *P* = 0.0006; Figure 4(d)). The post hoc analysis showed that this variable for the OVX was higher than for the C group (*P* < 0.01). The same was true for the comparison between the OVX + EB and C groups (*P* < 0.001). The percentage of neuritic AR-ir neurons was similar between the OVX and OVX + EB groups (*P* > 0.05).

### 3.6. GDNF and GFR*α*1 Expression

**Figure 5:**
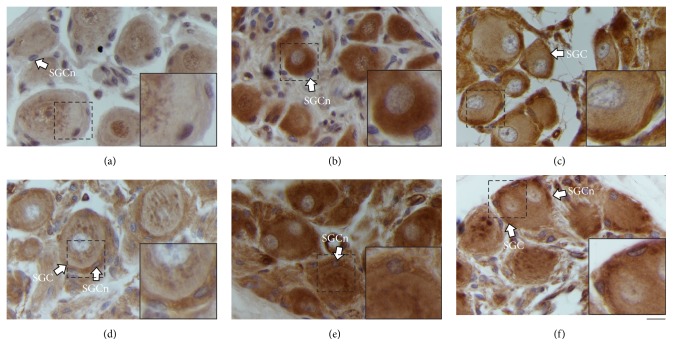
Expression of GDNF and GFR*α*1 in neurons and satellite glial cells (SGCs) of the paravaginal ganglia. Representative photomicrographs showing the GDNF (a–c) and anti-GFR*α*1 (d–f) in the paravaginal ganglia from the C (a, d), OVX (b, e), and OVX + EB (c, f) groups (*n* = 6 per group). Inset, magnification of fields indicated by* dashed squares*. SGC, satellite glial cell; SGCn, satellite glial cell nucleus. Bar, 20 *µ*m.

A cytoplasmic GDNF immunoreactivity was observed in paravaginal neurons for the C group (Figure 5(a)). This immunostaining was weaker than those for both OVX and OVX + EB groups (Figures 5(b) and 5(c)). Remarkably, a stronger GDNF immunoreactivity was observed around the peripheral nuclei attached to neuronal somata for the OVX + EB group, presumably belonging to the SGC (Figure 5(c)). The GFR*α*1 immunostaining was observed at cytoplasm of neurons for the C, OVX, and OVX + EB groups (Figures 5(d)–5(f)). This immunostaining for the OVX and OVX + EB groups was stronger than that for the C group (Figures 5(d)–5(f)). The GFR*α*1 immunoreactivity *α*was also observed in presumptive SGC being more intense for the OVX + EB than for the C and OVX groups (Figure 5(f)).

### 3.7. Satellite Glial Cells

**Figure 6:**
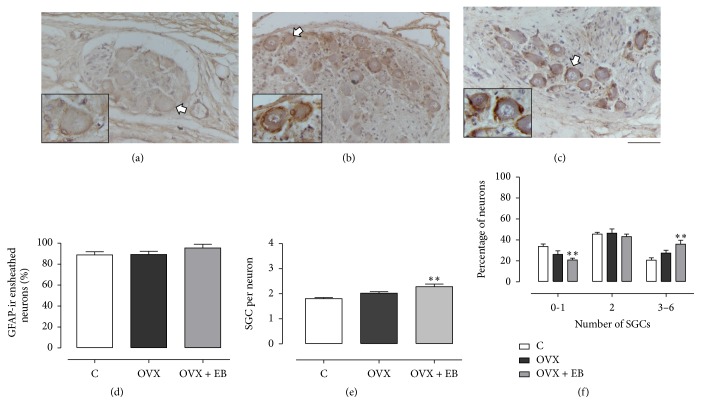
Satellite glial cells (SGCs) of the paravaginal ganglia. Representative photomicrographs showing the GFAP immunoreactivity surrounding neuronal somata for the C (a), OVX (b), and OVX + EB (c) groups. Inset, magnification of fields indicated by* dashed squares*. Percentage of GFAP-ir in the ensheathed neurons (d), number of SGCs per neuron as estimated from Masson-stained sections (e), and frequency distribution of attached SGC per neuron (f). Data are means ± SEM (*n* = 5 for both the C and OVX groups, and *n* = 6 for the OVX + EB group). One-way ANOVA followed by Newman–Keuls post hoc tests were carried out to determine significant differences between groups. ^*∗∗*^*P* < 0.01 (compared to the C group). Bar, 20 *µ*m.

The GFAP immunostaining was observed surrounding the somata of paravaginal neurons for the C (Figure 6(a)), OVX (Figure 6(b)), and OVX + EB groups (Figure 6(c)), supporting thus the assumed identity of the SGC. In comparison with the C group, a stronger intensity of the GFAP immunostaining in the SGC was found for the OVX and OVX + EB groups. The percentage of GFAP-ir ensheathed neurons was similar between the C, OVX, and OVX + EB groups (88.8 ± 3, 89.2 ± 3, 95.4 ± 3.6%;* F*_(2,12)_ = 1.302, *P* = 0.3079; Figure 6(d)). To estimate the number of SGCs per neuron, the peripheral nuclei adjoined to each neuronal soma were counted in the sections stained with Masson's trichrome (Figures 1(a)–1(c)). For this analysis, the number of sampled neurons was similar between groups (C, 63.6 ± 15.1; OVX, 55.8 ± 9.2; OVX + EB, 65.3 ± 18.4;* F*_(2,13)_ = 0.1074, *P* = 0.8989). As a result, the averaged number of SGCs per neuron was different between groups (C, 1.8 ± 0.04; OVX, 2 ± 0.05; OVX + EB, 2.2 ± 0.1;* F*_(2,13)_ = 9.816, *P* = 0.0025; Figure 6(e)). The post hoc analysis showed this variable was increased in the OVX + EB as compared to the C (*P* < 0.01) and OVX (*P* < 0.05) groups. The latter was associated with a high percentage of neurons surrounded by three or more SGCs (Figure 6(f)).

## 4. Discussion

Our present findings showed that the decrease in the soma size of neurons from paravaginal ganglia in OVX rabbits is related to the disturbance of the total serum E2 and T levels. This response supports the involvement of an increase in the extragonadal aromatization, which may occur even at the vagina. The administration of EB to ovariectomized rabbits was sufficient to preserve an averaged size of neuronal somata compared to control rabbits. This last finding is consistent with the effect of E2 administration on pelvic neurons of female rats but not on pelvic neurons of male rats [5, 11, 12].

Present findings support that a great extragonadal aromatization is linked to the recovery of serum E2 levels in chronically ovariectomized (5-month) rabbits [20]. As described for female rats [33], non-gonadal peripheral organs in which androgens are metabolized to estrogens should be further elucidated in rabbits. The expression of aromatase and ER in the vagina of monkeys and pelvic skeletal muscles of female rabbits supports that locally synthesized estrogen could influence the pelvic floor function [20, 23]. Findings herein extend this knowledge to the vagina of rabbits and, particularly, to paravaginal ganglia. Noteworthy, the increase in the expression of aromatase in paravaginal neurons, which may suggest an increment in the locally synthesized estrogens, was coincident with a decrease in the size of neuronal somata.

It is generally assumed that most of synaptic contacts in parasympathetic neurons are soma-dendritic [34]. If this were true for paravaginal neurons of rabbits, a decrease in their soma size could involve a loss of afferent inputs that may constitute an injury-like signal [35]. Certainly, low serum T levels have been related to the impairment of synaptic contacts in the soma of pelvic neurons [36]. Thus, future experiments should address this proposal. In this regard, the expression of aromatase in a high percentage of ganglion neurons could be considered as a neuroprotective response [34, 36, 37]. High serum E2 levels normalize the aromatase expression in paravaginal neurons in spite of the fact that low T levels persist in EB-treated rabbits in agreement with other findings [20, 33, 38, 39]. Moreover, it should be noted that the normalization of the aromatase expression in paravaginal neurons coincided with the recovery of the soma area.

The percentage of nuclear ER*α*-, ER*β*-, or AR-ir paravaginal neurons was not affected by the ovariectomy or the EB treatment. In contrast, the elevated percentage of neuritic AR-ir neurons for the OVX and OVX + EB groups was inversely related to total serum T levels. Because androgen actions influence the dendritic organization of pelvic neurons [12], a different arrangement of dendrites may be expected for neurons of both OVX and OVX + EB groups [11, 12, 40]. This proposal should be further explored approaching the density of dendrites and the complexity of their arborization. In this regard, estrogen actions could be also involved [12]. Taking into account the inverse relationship between the synaptic density of neuronal somata and dendrites [41], it would be relevant to evaluate whether the dendritic remodeling occurs prior to the recovery of the size of neuronal somata.

The intensity of cytoplasmic GDNF GFR*α*1 immunoreactivity observed in the paravaginal neurons for the OVX (heightened aromatase expression) and OVX + EB (high serum E2) groups suggests an upregulation by estrogens in accordance with other studies [24–26]. However, this was not sufficient to preserve the size of neuronal somata in OVX rabbits. The strong GDNF and GFR*α*1 immunostaining could be involved in the elaboration of a more complex dendritic organization of paravaginal neurons in the OVX and OVX + EB groups [42, 43], as supposed for the percentage of neuritic AR-ir neurons. Indeed, such hypothesis should be tested approaching the organization of dendrites of paravaginal neurons. In addition, it would be also worthy to explore other neurotrophins and their receptors that could be involved in the estrogen-induced plasticity of neuronal soma of pelvic neurons [16, 44]. Otherwise, our findings suggest that the preservation of the neuronal soma size requires an increase in the GDNF of the SGC.

Despite the fact that a few of studies have approached the relevance of the SGC in the parasympathetic ganglia, the number of SGCs has been related to the size of neuronal somata and the synaptic organization during the growth and across lifespan [29, 45–47]. Furthermore, the postnatal addition of the SGC is linked to the generation of more synaptic buttons in the parasympathetic neurons [29]. In agreement with these studies, our present findings suggest that the preservation of the size of neuronal soma promoted by estrogens requires the activation (stronger GFAP-ir) and, likely, proliferation of the SGC, as well as an increase in the GDNF expression. Indeed, the SGC could favor the overexpression or post-processing of the GDNF as reported for other neurotrophic factors [48, 49]. Remarkably, the SGCs express ER ability to modulate the expression of several genes as a response of E2 treatment [50]. Moreover, estrogens can modulate the proliferation and/or activation of other glial cells in the peripheral as well as in the central nervous system [51].

The averaged number of SGCs per neuron agrees with studies focused on the relationship between neuron and SGC in autonomic ganglia of mice [29, 45] and sheep [46]. Indeed, the study reported by Pomeroy and colleagues [29] along the postnatal development of mice, even at adulthood, carried out a correction factor to estimate the number of SGCs per neuron given as a result approximately two SGCs per neuron. Since the number of SGCs per neuron was estimated based on the counting of a single section herein, which may imply missing SGCs in regions of those neurons not included in the section, we also evaluated the frequency distribution of neurons having a different number of SGCs. To do this, we observed a high percentage of neurons surrounded by at least three SGCs (with a maximum of six SGCs per neuron) for the OVX + EB group. Certainly, a large neuronal soma area is related to a great number of associated SGCs [29]. Unfortunately, data regarding the pair of area of neuronal somata and number of SGCs were not gathered in our present study and the respective correlation was not determined.

Pelvic neurons comprise a heterogeneous population as supported by studies conducted in other mammal species [1]. For the case of paravaginal ganglia, it has been reported that most of their neurons are cholinergic (ChAT-positive) [7] that, likely, receive an important input from pelvic nerves [52]. Therefore, it is plausible to propose that parasympathetic neurons are more affected by the estrogenic status than sympathetic ones. Nevertheless, this proposal should be addressed in future studies.

Changes in the size of pelvic neurons may alter the threshold of reflexes and visceromotor functions of urogenital organs [53]. Indeed, from paravaginal ganglia arise fibers toward genitourinary organs and rectum [52]. Furthermore, ovariectomy in rabbits reduces the vaginal blood flow in response to pelvic nerve stimulation, which is clearly recovered by E2 and, in a lesser extent, by T [54]. These effects seem to be mediated by the fact that both hormones regulate the endothelial nitric oxide synthase [54]. Since most of paravaginal neurons are cholinergic [7], it could be speculated that the reduction in the neuronal size is linked to the impairment of the vaginal blood flow. Certainly, future experiments should identify other molecules participating in the vasodilation (i.e., NOS, VIP, NPY, and CGRP). Moreover, specific targets of paravaginal neurons should be identified to determine specific physiological changes.

The hormonal milieu is a source of variability for the pelvic plexus that influences the structure and, likely, function of neurons and SGC. Indeed, pregnancy has a clear impact on the size of neuronal somata of paravaginal ganglia in rabbits [7] as compared to that of the vaginal distention achieved in non-cycled female rats [55]. Taking into account that the organization of SGC changes along lifespan [29, 45] and its trophic involvement in the neuronal morphology [48, 49], our present study highlights a plausible contribution of the SGC in the estrogen-related plasticity of the somata size of paravaginal neurons. Further experiments should determine particular associated processes (i.e., variations in neuronal metabolism, synaptic and/or dendritic organization, profile of neurotransmitters and neuromodulators, and expression of markers of glial activity). This could be insightful to elaborate novel hypotheses regarding the origin of some urogynecological symptoms across the life of women, for instance, at pregnancy and menopause, in which pelvic plexus alterations are involved [3, 4]. Future studies should evaluate other ganglia related to the female LUT, including ganglia of the sympathetic chain and dorsal root ganglia.

## 5. Conclusions

Our findings demonstrate that chronic ovariectomy reduces the size of neuronal somata of paravaginal ganglia, which is related to an increase in the extragonadal aromatization as supported by the E2/T ratio. Particularly, the high percentage of neurons expressing aromatase supports the relevance of local estrogen synthesis for paravaginal neurons. The decrease in the neuronal size was further related to an elevated percentage of neuritic AR-ir neurons and a high expression of GDNF and GFR*α*1 in neurons. The increase in the serum E2 levels due to the EB treatment prevents the decrease in the size of neuronal somata, which is linked to a normalization of the aromatase expression in neurons, an increase in the expression of GDNF in the SGC, and an increment in the SGC per neuron.

## Figures and Tables

**Table 1:** Primary and secondary antibodies and normal sera used in the present study.

Antibody	Dilution	Catalogue number	Manufacturer
Rabbit polyclonal IgG anti-aromatase	1 : 500	NB-200-1596	Novus Biologicals
Mouse monoclonal IgG anti-ER*α*	1 : 150	MA3-310	Thermo Scientific
Mouse monoclonal IgG anti-ER*β*	1 : 150	MA1-23217	Thermo Scientific
Rabbit polyclonal IgG anti-AR	1 : 500	sc-816	Santa Cruz Biotechnology, Inc.
Mouse monoclonal IgG anti-GDNF	1 : 100	sc-13147	Santa Cruz Biotechnology, Inc.
Mouse monoclonal IgG anti-GFR*α*1	1 : 100	sc-271546	Santa Cruz Biotechnology, Inc.
Goat polyclonal IgG anti-GFAP	1 : 100	sc-6170	Santa Cruz Biotechnology, Inc.
Goat anti-mouse IgG-biotinylated	1 : 250	sc-2039	Santa Cruz Biotechnology, Inc.
Goat anti-rabbit IgG-biotinylated	1 : 2000	sc-2040	Santa Cruz Biotechnology, Inc.
Donkey anti-goat IgG-biotinylated	1 : 250	sc-2042	Santa Cruz Biotechnology, Inc.
